# Langerhans Cell Histiocytosis of the Uvea with a Ciliochoroidal Mass: A Case Report of Management with Systemic Therapy

**DOI:** 10.1155/2023/5543131

**Published:** 2023-09-01

**Authors:** Fariba Ghassemi, Hamid Riazi-Esfahani, Nazanin Ebrahimiadib, Abdulrahim Amini, Zahra Mahdizad

**Affiliations:** ^1^Eye Research Center, Farabi Eye Hospital, Tehran University of Medical Sciences, Tehran, Iran; ^2^Retina & Vitreous Service, Farabi Eye Hospital, Tehran University of Medical Sciences, Tehran, Iran; ^3^Department of Ophthalmology, University of Florida, Gainesville, FL, USA; ^4^Department of Ophthalmology, School of Medicine, Hormozgan University of Medical Sciences, Bandar Abbas, Iran

## Abstract

**Background:**

This study is aimed at exploring a case of choroidal Langerhans cell histiocytosis (LCH) successfully treated with systemic corticosteroid and immunosuppressant. *Case presentation*. A 24-year-old man with known multisystem LCH developed loss of vision, ocular pain, conjunctival injection, panuveitis, and a ciliochoroidal mass. After receiving an intravenous methylprednisolone pulse, oral high-dose corticosteroids, and methotrexate, the mass resolved quickly and completely without flare-ups during 6 months of follow-up.

**Conclusions:**

Intraocular involvement of LCH is rare and can present with or without a history of multisystemic disease. The diagnosis is challenging, and the standard treatment is not established. Systemic anti-inflammatory and immunosuppressive therapy could be an effective treatment, as the LCH itself contains an essential element of inflammation and the symptoms may be mainly inflammatory.

## 1. Background

Langerhans cell histiocytosis (LCH) is the most common histiocytic disorder characterized by the accumulation of histiocytes in various tissues [1]. It includes a broad spectrum of clinical presentations, ranging from self-healing lesions to life-threatening disseminated disease [1]. The disorder is most common in children (peaks between ages 1 and 4), but anyone can be affected [2]. Although there is strong evidence for an underlying inflammatory process [3], a recent mutation found in LCH patients of BRAF-V600E makes the cancerous etiology more clear [1, 4]. Therefore, some may consider LCH to be inflammatory myeloid neoplasia [5].

LCH can be diagnosed by clinical and paraclinical findings, but in particular by histopathological analyses demonstrating the presence of histiocytes with specific characteristics of Langerhans cells (LCs) [6]. More frequently affected organs are the bones, skin, pituitary gland, liver, spleen, hematopoietic system, and lungs [1]. Most cases of LCH present with ophthalmic involvement, namely, orbital involvement, whereas intraocular involvement is rare [3, 7].

## 2. Case Presentation

A 24-year-old man was referred to our oncology clinic for evaluation of an amelanotic mass in his left eye. His symptoms have included pain, redness, proptosis, and visual loss in his left eye, as well as nasal congestion and posterior nasal drip, over the past few months. About 3 months before, he underwent ethmoid and maxillary sinus biopsies, which were reported to be a nonspecific orbital inflammation (NSOI) with extension to the sinuses based on the presence of only CD34-positive cells. While the patient was treated with systemic steroids, his symptoms (especially the loss of vision) did not improve significantly. The patient presented with a history of bilateral anterior uveitis in remission in addition to juvenile idiopathic arthritis (JIA). His history also included multisystemic LCH since childhood, which was diagnosed based on his symptoms (lymphadenopathy, skin eruptions, frontal bone lesions, sinusitis, epilepsy, and diabetes insipidus) and the presence of CD1a-positive cells in lymph node specimens. He received systemic prednisolone and vinblastine, which were tapered upon improvement of his condition. At the time of presentation, he was taking oral prednisolone 50 mg daily, valproic acid 500 mg daily, eye drop betamethasone QID, and eye drop homatropine TDS.

**Figure 1:**
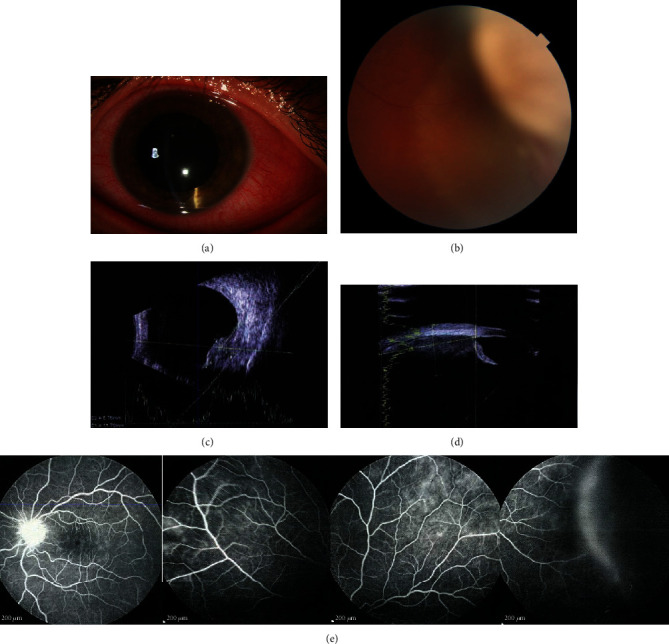
A 24-year-old man presented with conjunctival injection (a), pain, and visual loss. He was found to have an amelanotic choroidal mass (b) and panuveitis. Ultrasound evaluation using B-scan demonstrated a dome-shaped mass located temporally, measured 12^∗^ 9^∗^6.7 mm, with primarily high internal reflectivity and a mound of tissue with variable reflectivity and a well-bordered mass inside the main lesion. (c) In UBM, the mass had variable internal reflectivity with cystic alterations and spread anteriorly to the iris root (d). Fluorescein angiography revealed optic nerve head leakage, posterior and peripheral vasculitis, and staining of the lesion (e).

His best-corrected visual acuity (BCVA) in the right eye (OD) was 10/10, while in the left eye (OS), it was 6/10. On the temporal side of OS, diffuse conjunctival injection with localized scleral and episcleral thickening were found with dilated (sentinel) vessels on the temporal side (Figure 1(a)). 2+ anterior chamber (AC) and anterior vitreous (AV) reactions were also detected. In the far peripheral region of the superotemporal area of the retina, a subretinal creamy-white mass with a cuff of subretinal fluid (SRF) was found (Figure 1(b)). On ultrasonography, a choroidal mass with a diameter of 12 × 9 × 6.7 mm was seen with a primarily high internal reflectivity and a mound of tissue with variable reflectivity and a well-bordered mass inside the main lesion that extended anteriorly to the ciliary body (Figures 1(c) and 1(d)).

Fluorescein angiography (FA) in OD was unremarkable, while in OS, it showed posterior and peripheral vasculitis, as well as leakage from the optic disc. (Figure 1(e)).

The diagnosis of unilateral panuveitis with anterior scleritis and inflammatory choroidal mass was made. The systemic workup for rheumatologic disease and granulomatosis disease came back negative, and the medical examination revealed no other systemic focus of disease activity. Ibuprofen (400 mg QID) and methotrexate (15 mg weekly) were administered. In the following follow-up sessions by 1 month, both his symptoms and the size of the choroidal lesion decreased substantially.

**Figure 2:**
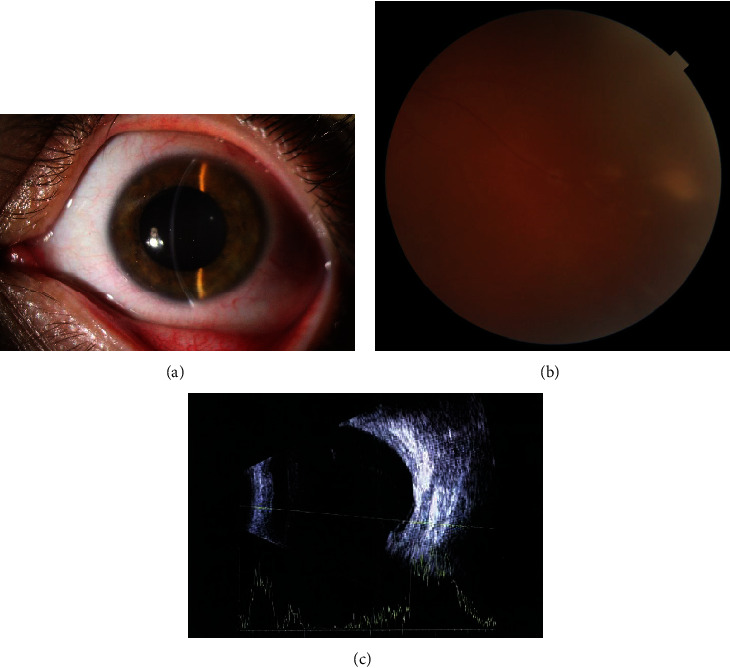
Two weeks following therapy, slit-lamp examination revealed reduced conjunctival injection and scleral edema (a), fundus examination demonstrated significant decrease in mass size and subretinal white deposits with trace SRF at the site of resolved mass (b), and b-scan also showed resolution of choroidal mass with small amount of subretinal fluid (c).

A flare-up occurred two months after the initial presentation. The BCVA of OS was counting finger at 3 meters, and the examination revealed conjunctival injection, chemosis, hazy media, and AC and AV inflammation (3+) along with a larger mass (15 × 11.5 × 9.7 mm). He was admitted with the diagnosis of LCH-associated panuveitis and choroidal mass. Intravenous methylprednisolone pulse was administered for 3 days, and he was discharged with prescription of oral prednisolone 50 mg daily, methotrexate 15 mg weekly, and Ibuprofen 400 mg every 4 hours. The pain, conjunctival injection, and SRF amount decreased in a few days, and after two weeks, his BCVA improved to 2/10, the AC and AV inflammation decreased to trace cells, and the mass size decreased significantly as it could hardly be seen in the fundus examination and B-scan. Subretinal white deposits with trace SRF at the site of resolved mass were visible (Figures 2(a)–2(c)). In addition to discontinuing the Ibuprofen, the dosage of the corticosteroid medication was tapered cautiously. The patient has been followed for the last six months with a stable condition, and his vision has improved to 9/10.

## 3. Discussion and Conclusions

LCH is a rare systemic disorder characterized by the proliferation of clonal S-100, CD1a, and CD207-positive Langerhans cells in a wide range of organs [8]. Due to its myeloid origin and inflammatory characteristics, it has been considered a myeloid neoplasm [5]. A number of mutations, including BRAF (the most common one), MAP2K1, and ARAF, have been identified in LCH patients, all of which activate the MAPK/ERK signaling pathway [9].

A typical pathologic histiocyte in LCH consists of mononucleated cells with indented or kidney-shaped nuclei and a Birbeck granule; however, in recent years, the search for CD1a and CD207 (Langerin) expression has largely replaced Birbeck granule detection [6, 10]. Furthermore, BRAF V600E mutation analysis in plasma and urine has shown to be an effective diagnostic tool [9].

LCH may affect any organ of the body (individually or in conjunction). The disease is classified clinically as single-system Langerhans cell histiocytosis (SS-LCH) or multisystem Langerhans cell histiocytosis (MS-LCH) with multiple organ involvement. The hematopoietic system (bone marrow), spleen, and liver are also at risk [8]. Isolated pulmonary LCH is the most common manifestation in adults, but diabetes insipidus (DI) is the most prevalent initial sign of central nervous system involvement in LCH.

Intraocular involvement has mostly been reported in case reports [7]. The choroid is the most common intraocular site of manifestation, and it may be infiltrated diffusely or as a solitary mass [11]. Although there have been reports of abnormal LCs infiltrating the sclera, retina, vitreous, uveal tract, and optic nerve sheath in patients with and without a history of multisystemic LCH, this is a rare occurrence [10–12].

The treatment of LCH depends on its heterogeneous presentation; generally, patients with a single-system disease only need local treatment (e.g., radiotherapy) or observation, while those with a more extensive disease require systemic therapy [9]. The standard treatment for MS-LCH has yet to be identified, but vinblastine and prednisolone therapy have been suggested (as with our patient); short-term chemotherapy with MTX, doxorubicin, cyclophosphamide, vincristine, and bleomycin, as well as allogeneic hematopoietic stem cell transplantation, have shown efficacy in more aggressive cases [8]. Clinical trials of targeted therapies with selective BRAF and MEK1/2 inhibitor medications are ongoing and seem to be effective [8, 9].

Our patient presented at the age of 24 with unilateral panuveitis, scleritis, and choroidal mass refractory to the treatment with oral prednisolone. Considering his unremarkable laboratory work-up and systemic disease, we concluded that MS-LCH was the cause of his ophthalmic symptoms. Similar cases of choroidal LCH presenting as unilateral choroidal masses, panuveitis, or a combination of both have been reported (Table 1) [7, 10, 12–14]. In one case, a diagnosis of choroidal melanoma was made, but immunohistochemical studies revealed LCH [10], and in the second eye, the iris portion of an iridociliochoroidal mass was found to be LCH after fine-needle aspiration [7]. Some reported vitreous biopsy for diagnosis of intraocular LCH [15], whereas others made the diagnosis based on a systemic history of LCH [11, 16, 17], as we did for our patient.

Considering the rare incidence of intraocular involvement of LCH, there is no standard treatment. Patton et al. [13] reported the first case of choroidal involvement as a mass by LCH and documented successful outcomes of fractionated low-dose, whole-eye external beam radiotherapy for choroidal LCH. Shields et al. [7] used brachytherapy for treatment of an iridociliochoroidal tumor in a 6-year-old boy with known MS-LCH. The tumor responded rapidly with complete regression.

As LCH itself contains an essential element of inflammation, and our patient had inflammatory symptoms and a first response to systemic corticosteroid, we decided to continue with high-dose corticosteroids (intravenous methylprednisolone pulse) in combination with immunosuppression (methotrexate), which proved to be effective. To the best of our knowledge, this is the first case of choroidal mass associated with LCH successfully treated with systemic corticosteroid and immunosuppressant without radiotherapy.

## Figures and Tables

**Table 1:** Reported cases of choroidal Langerhans cell histiocytosis mass.

No.	Authors	Year	Age	Sex	Presentation	Tumor size	Diagnosis method	Treatment	Outcome	Ref.
1	Shields et al.	2010	6	M	Iridociliochoroidal mass and neovascular glaucoma	A 14 mm in basal diameter and 8.4 mm in thickness	Fine needle aspiration biopsy	Low-dose plaque radiotherapy of	Complete regression to a flat scar of 2 mm thickness and no sign of radiation retinopathy	[7]
2	Kim and Lee	2000	49	M	Choroidal mass resembling choroidal melanoma	6 DD	Tumor biopsy	Enucleation (with a diagnosis of choroidal melanoma)	—	[10]
3	Narita et al.	1993	61	F	A solitary massive tumor and recurrent anterior uveitis		Biopsy	Enucleation		[12]
4	Patton et al.	2006	29	M	Choroidal mass	5 DD, 2 mm thickness	Patient history of central nervous system Langerhans cell histiocytosis lesion	Low-dose external fractionated radiotherapy	A rapid improvement in both visual symptoms and clinical appearance	[13]
5	Angell and Burton	1978	5	F	Choroidal mass and secondary retinal detachment	1.75 mm	Patient history of MS-LCH	—	—	[14]
6	Ghassemi et al.	2023	24	M	Choroidal mass, panuveitis, and scleritis	15 × 11.5 × 9.7 mm	Patient history of MS-LCH	Intravenous methylprednisolone pulse followed by oral high-dosage corticosteroid and methotrexate	Rapid and complete resolution	

## Data Availability

The datasets used during the current study are available from the corresponding author on reasonable request.

## Abbreviations

LCH: Langerhans cell histiocytosis
LCs: Langerhans cells
NSOI: Nonspecific orbital inflammation
JIA: Juvenile idiopathic arthritis
CD: Cluster of differentiation
QID: Quater in die
BCVA: Best-corrected visual acuity
OD: Oculus Dexter
OS: Oculus sinister
AC: Anterior chamber
AV: Anterior vitreous
SRF: Subretinal fluid
FA: Fluorescein angiography
SS-LCH: Single-system Langerhans cell histiocytosis
MS-LCH: Multisystem Langerhans cell histiocytosis
DI: Diabetes insipidus.
